# Characterization of novel dual tandem CD19/BCMA chimeric antigen receptor T cells to potentially treat multiple myeloma

**DOI:** 10.1186/s40364-020-00192-6

**Published:** 2020-05-13

**Authors:** Liqing Kang, Jian Zhang, Minghao Li, Nan Xu, Wei Qi, Jingwen Tan, Xiaoyan Lou, Zhou Yu, Juanjuan Sun, Zhenkun Wang, Chengcheng Fu, Xiaowen Tang, Haiping Dai, Jia Chen, Depei Wu, Lei Yu

**Affiliations:** 1 Institute of Biomedical Engineering and Technology, Shanghai Engineering Research Center of Molecular Therapeutics and New Drug Development, School of Chemistry and Molecular Engineering, East China Normal University, NO, 3663 North Zhongshan Road, Shanghai, 200065 China; 2 National Clinical Research Center for Hematologic Diseases, Jiangsu Institute of Hematology, The First Affiliated Hospital of Soochow University, Suzhou, China; 3 Institute of Blood and Marrow Transplantation, Collaborative Innovation Center of Hematology, Soochow University, Suzhou, China; 4 Shanghai Unicar-Therapy Bio-medicine Technology Co., Ltd, No 1525 Minqiang Road, Shanghai, 201612 China; 5 Central Laboratory of Hematology and Oncology, First Affiliated Hospital, Harbin Medical University, Harbin, 150001 Heilongjiang Province China

**Keywords:** Tandem-CAR T, Multiple myeloma, CD19, BCMA, Relapse

## Abstract

**Background:**

Treatment with chimeric antigen receptor (CAR)-engineered T cells directed against the B-cell maturation antigen (BCMA) promoted transient recovery from multiple myeloma (MM). However, the absence of this antigen on immature plasma cells may limit the efficacy of this modality and facilitate relapse. The purpose of this study is to characterize a novel CAR that includes both a single-chain variable fragment (scFv)-BCMA and an scFv-CD19 in tandem orientation (tan-CAR) in an attempt to target both BCMA and CD19 expression on MM cells.

**Method:**

The scFv sequences from the anti-CD19 antibody FMC63 and the anti-BCMA antibody C11D5.3 were ligated in tandem with transmembrane and T-cell signaling domains to generate the tan-CAR construct. Specificity and efficacy of activated tan-CAR T cells were analyzed using in vitro proliferation, cytokine release, and cytolysis assays. We also evaluated the in vivo efficacy with a xenograft mouse model that included target tumor cells that expressed CD19 or BCMA and compared the results to those obtained with conventional CAR T cells.

**Results:**

The in vitro studies revealed specific activation of tan-CAR T cells by K562 cells that overexpressed CD19 and/or BCMA. Cell proliferation, cytokine release, and cytolytic activity were all comparable to the responses of single scFv CAR T cells. Importantly, in vivo studies of tan-CAR T cells revealed specific inhibition of tumor growth in the mouse xenograft model that included cells expressing both CD19 and BCMA. Systemic administration of tan-CAR T cells resulted in complete tumor remission, in contrast to the reduced efficacies of BCMA-CAR T and CD19-CAR T alone in this setting.

**Conclusion:**

We report the successful design and execution of novel tan-CAR T cells that promote significant anti-tumor efficacy against both CD19 and BCMA antigen-positive tumor cells in vitro and in vivo. The data from this study reveal a novel strategy that may help to reduce the rate of relapse in the treatment with single scFv-CAR T cells.

## Introduction

Multiple myeloma (MM) is a malignant neoplasm in which uncontrolled expansion and proliferation of clonal plasma cells leads to osteolytic lesions and bone marrow failure in association with end-organ damage [1]. Several new drugs and drug regimens have recently been introduced in an effort to improve treatment for MM. Although these regimens are overall safer than previous therapies, only a limited number patients respond completely and effectively [2–4]. As such, we need to consider more innovative strategies with the aim of generating a more significant and long-lasting therapeutic effect.

Cellular immunotherapy is a novel and evolving treatment strategy in which cytotoxic T cells are engineered to promote recognition of specific tumor antigens. Adoptive transfer of chimeric antigen receptor (CAR)-engineered autologous T cells has met with unprecedented success for the treatment of hematological malignancies [5–7]. In parallel, several diverse immunotherapeutic approaches currently under investigation have utilized this approach and focus on engineering target antigen specificity and T-cell activation [8]. The CAR T-cell approach for the treatment of MM has shown considerable promise and has been associated with manageable toxicities. Notably, several efforts have focused on B-cell maturation antigen (BCMA) due to its preferential expression on plasma cells [9–11]. To date, early phase clinical trials that explore the impact of single-chain fragment variable (scFv) anti-BCMA-modified CAR T cells have shown undeniably high response rates. Unfortunately, the responses are often transient with frequent relapse [12]. One of the reasons of relapse might due to a group of residual malignant CD19^+^ plasma cells which can be detected among the tumor cells; these cells can drive self-renewal, myeloma propagation, and resistance to chemotherapy and can be considered to be cancer stem cells [13]. Furthermore, sustained remission was observed with advanced MM in one patient who received anti-CD19 CAR T cells in conjunction with an autologous stem cell transplantation [14]. Thus, CD19 might be the potential target for multiple myeloma treatment. Moreover, sequential delivery of BCMA-CAR and CD19-CAR T cells resulted in a strong therapeutic outcome; preliminary data suggested that amplification of CD19-CAR T cells might be critically associated with this response and even the absence of even minimal residual disease [15]. However, it is critical to note that patients diagnosed with associated lymphocytopenia may not have enough T cells for the production of two CAR T products; high manufacturing costs are also a key limitation to be considered. We also note that sequential delivery of two independent CAR T products might be associated with limited efficacy of the second infusion [16]. Previous study proved bi-specific CAR capable of preventing antigen escape in vivo by post-mortem analysis which revealed the outgrowth of CD19− mutants in the mixed-Raji xenograft [17]. Taken together, these results suggest that we might employ CAR T cells that simultaneously recognize both CD19 and BCMA for effective treatment of MM and reduce the risk of relapse.

Here, we describe a novel CAR lentiviral construct with tandem alignment of a dual scFv (tan-CAR) targeting both CD19 and BCMA antigens. To the best of our knowledge, this is the first time this approach has been considered. Among our results, we found that tan-CAR T cells targeting one or both antigens promote equivalent cytotoxic effects in vitro as do conventional CAR T cells with only a single scFv. Interestingly, the results of our in vivo studies suggested that tan-CAR T cells were capable of eradicating a mix of malignant cells that express CD19 or BCMA, ultimately resulting in complete remission. As such, our results suggest that the tandem-dual antigen targeting strategy will represent effective anti-neoplastic therapy may ultimately prevent relapse secondary to absence or loss of BCMA expression on the malignant MM cells.

## Methods

### Plasmid construction and production of recombinant lentiviral vectors

The tandem-CAR construct is a second-generation vector consisting of the following components in-frame from the 5′ end to the 3′ end: the CD8 signal peptide sequence, anti-BCMA-scFv (C11D5.3) [18]_,_ anti-CD19 scFv (FMC63AA 1–267, GenBank ID: HM852952.1), the hinge and transmembrane regions of the CD8α molecule, the cytoplasmic domain of CD28, and the CD3 zeta signaling domain. The sequence was synthesized by Tsingke Biological Technology (Shanghai, China) and cloned into the pUT plasmid backbone (Unicar-Therapy Biomedicine Technology Co., Ltd., Shanghai, China). The newly-constructed lentiviral vector is referred to as tan-CAR. We also prepared the scFv domain CAR vectors CD19-CAR and BCMA-CAR with the CD8 signal peptide sequence anti-CD19 scFv (FMC63AA 1–267, GenBank ID: HM852952.1) or anti-BCMA-scFv (C11D5.3), the hinge and transmembrane regions of the CD8α molecule and cytoplasmic domain of CD28 and the CD3 zeta signaling domain. Lentiviruses were generated from these constructs via transient transfection of HEK293T cells.

### Cell lines

All the cell lines were purchased from the American Tissue Culture Collection (Manassas, VA, USA) and cultured in Roswell Park Medical Institute (RPMI)-1640 medium supplemented with 10% heat-inactivated fetal bovine serum (FBS; HyClone, Logan, UT, USA). K562 cells were stably transduced with the lentiviral constructs encoding CD19 or BCMA and luciferase. Following transduction, single luciferase-positive cells were selected for clonal expansion. K562-CD19-luc and K562-BCMA-luc stable cell lines were generated by this method. Myeloma cell line 8226 were transduced with the lentiviral constructs encoding CD19 to obtain the tumor cells expressing both CD19 and BCMA.

### Preparation of CAR T cells

Healthy donor-derived peripheral blood mononuclear cells were isolated from blood by gradient centrifugation using Lymphoprep™ (Oriental Hua Hui, Beijing, China) followed by CD3^+^T-cell enrichment by positive selection using a magnetic bead separation method (Miltenyi Biotec, Bergisch Gladbach, Germany). CD3^+^T cells were cultured and activated in vitro using anti-CD3/CD28 monoclonal antibodies (Miltenyi Biotec) in a 5% CO_2_ atmosphere at 37 °C for 18–24 h. The activated T cells were then transduced with lentivirus (CD19-CAR, BCMA-CAR and tandem CAR) for 48 h. We also tansduced the BCMA CAR (D1) followed by CD19-CAR (D2) to obtain the T cells expressed two scFv by transduced two lentivirus. After transduction, the CAR T cells were cultured and expanded in a 5% CO_2_ atmosphere at 37 °C for 14 days in AIM-V medium (Gibco, Grand Island, NY, USA), supplemented with 100 IU/mL recombinant human interleukin-2 (IL-2; Peprotech, Rocky Hill, NJ, USA), 5 ng/ml recombinant human IL-7 (Peprotech), 5 ng/mL recombinant human IL-15 (Peprotech) and 10% autologous plasma [19].

### Flow cytometry

For the flow cytometry assays, the cells were harvested and washed twice with 1 mL of phosphate-buffered saline (PBS) containing 2% FBS (Gibco). To determine transduction efficiency and the CD4/CD8 ratio, the CAR T cells were labeled with the recombinant protein L-FITC (ACRO Biosystems, Beijing, China), anti-CD4-PE-Cy7 (eBioscience, San Diego, CA), and anti-CD8-APC (eBioscience) for 45 min at 4 °C in the dark. For detection of the CD19 CAR-expressing T cells, the CAR T cells were incubated with human CD19 protein-FITC (ACRO) for 45 min at 4 °C in the dark. For detection of the BCMA-CAR T cells, the CAR T cells were labeled with human BCMA protein-FITC (ACRO) for 45 min at 4 °C in the dark. The cells were washed twice before analysis by Attune NxT flow cytometer (Thermo Fisher, Waltham, USA).

### T-cell activation assay

T-cell activation was evaluated by measuring CD69 expression on tan-CAR T cells in response to 24-h co-culture with target cells. Un-transduced (NC) T cells were used as negative controls, and the T cells transduced with CD19-CAR or BCMA-CAR served as positive controls. After co-culture, the cells were harvested and washed twice with 1 mL of PBS containing 2% FBS and then labeled with CD69-PE (Biolegend, San Diego, CA, USA), CD3-FITC (Biolegend), and protein L-FITC (ACRO) for 20 min at room temperature in the dark. CD69 expression in CAR T cells as detected by flow cytometry was used as a marker of CAR T-cell activation.

### Quantitation of T-cell proliferation

Cell proliferation assays were performed using a Carboxyfluorescein Diacetate Succinimidyl Ester (CFSE) assay kit (Abcam, Cambridge, UK) following the manufacturer’s instructions. In brief, the CAR T cells were labeled with 2.5 μM CFSE and then co-cultured with Raji cells which treated with mitomycin before to stop the division, at a stimulator to responder ratio of 2:1 (10^6^ CAR T cells/mL) for 5 days in 96-well plates in 200 μL serum-free AIM-V (Gibco) medium per well. Flow cytometry was performed using an Attune NxT flow cytometer (Thermo Fisher) to detect changes in CFSE intensity. FlowJo V10 software (TreeStar, San Carlos, CA, USA) was used for data analysis.

### Cytotoxicity assays

Cytotoxicity was determined via quantitation of lactate dehydrogenase activity in the supernatants of effector and target cell co-cultures using the Cytotoxicity Detection Kit (Promega, Madison, WI, USA) following the manufacturer’s protocol. All the transduced CAR T cells (effector, E) were co-cultured with cells of the target cells that overexpressed CD19, BCMA, or both antigens (target, T) at E:T ratios of 5:1, 2.5:1, and 1:1, respectively. Target and effector cells were seeded in 96-well plates in a total volume of 100 μL of serum-free RPMI 1640 media (Gibco) and incubated at 37 °C for 6 h. After co-culture, 50 μL of cell-free supernatant from each well was transferred to a new 96-well plates and mixed with equal volume of lactic acid dehydrogenase substrate mixture for 20 min at room temperature in the dark. The absorbance was recorded at 492 nm using a full wavelength reader Multiskan GO (Thermo Scientific). Tumor (target cell) lysis was calculated with the following formula: % lysis = (experimental LDH release − spontaneous LDH release) / (maximum LDH release − spontaneous LDH release) × 100.

### Detection of CD107a

To evaluate CD107a expression on the cell surface as an indirect marker of degranulation, 10^6^ CAR T cells were co-cultured with target cells at a 5:1 ratio in 96-well plates with a total of 200 μL of AIM-V (Gibco) medium per well for 6 h. The Golgi inhibitor monensin (Invitrogen, Carlsbad, CA, USA) was added before the incubation. Cocktails (Invitrogen) were added to the positive control group before co-culture. After a 6-h incubation, cells were labeled with anti-CD107a-APC, anti-CD3-FITC, and anti-CD8-PE. All the antibodies were purchased from Becton, Dickinson and Company Co., Ltd. (Franklin Lakes, NJ, USA). Cells were collected, washed twice with PBS, and flow cytometry analysis was performed on an Attune NxT flow cytometer (Thermo Fisher). The results were analyzed by FlowJo V10 software (TreeStar).

### Analysis of cytokine release

Cytokine release was evaluated using a Th1/Th2 Cytometric Bead Array (CBA) Kit II (BD Bioscience) according to the manufacturer’s instructions. Briefly, CAR-transduced T cells were co-cultured with the various K562 cell transfectants at an E:T ratio of 5:1 in a 96-well plate with a total volume of 200 μL of RPMI 1640 medium (Gibco). After 24 h in co-culture, cell-free supernatants were harvested and the levels of various cytokines were evaluated. The capture microspheres for seven specific cytokines (IL-2, IL-4, IL-6, IL-10, IFN-γ, TNF-a, and IL-17A) were first mixed and then incubated with the sample and fluorescent antibody for 3 h. The mixture was washed and cytokine concentrations were determined by flow cytometry (Thermo Fisher). The concentration of each cytokine was calculated from standard curves.

### Mouse xenograft model

Mouse experiments were performed with the approval of the Institutional Animal Care and Use Committee of East China Normal University. Four-to-six-week-old male NOD/scid/γc^−/−^ (NSG) mice were purchased from Biocytogen Co., Ltd. (Beijing, China). Xenograft models were established via injection of K562-CD19-luc and/or K562-BCMA-luc cells. A total of 7 × 10^6^ mixed tumor cells at a ratio of 1:1 in 200 μL PBS were injected into mice via the tail vein on day 0. The mice were then randomly divided into four groups that received either (a) 10^7^ CD19-CAR T cells (*n* = 3), (b) 10^7^ BCMA-CAR T cells (n = 3), (c) 10^7^ tan-CAR T cells (n = 3) or (d) 10^7^ un-transduced T cells (n = 3, negative control) via the intravenous route on both days 8 and 10. The mice injected with K562-CD19-luc or K562-BCMA-luc were treated with CD19-CAR T or BCMA-CAR T cells, respectively. Tumor progression was monitored by bioluminescent imaging every 4 days beginning on day 7. The mice were sacrificed when moribund or upon the development of hind-limb paralysis. For in vivo imaging, the mice were injected intraperitoneally with 150 mg/kg D-luciferin (Yeasen, Shanghai, China) and imaged under isoflurane anesthesia using the Xenogen-IVIS system (LI-COR Biosciences, Lincoln, NE, USA). Fluorescence was quantified using Living Image software (IVIS Lumina Series, PerkinElmer, Waltham, MA, USA).

### Statistical analysis

Statistical analyses were carried out using GraphPad Prism 8.0. Biological replicates of in vitro (*n* = 3) and in vivo data (n = 3) are presented as the mean ± SD. Statistical analysis was performed to assess differences between individual treatment groups and the un-transduced control group using one-way ANOVA. Statistically significant findings were defined as *p < 0.05.

## Results

### Generation and characterization of the tan-CAR-transduced T cells

**Fig. 1:**
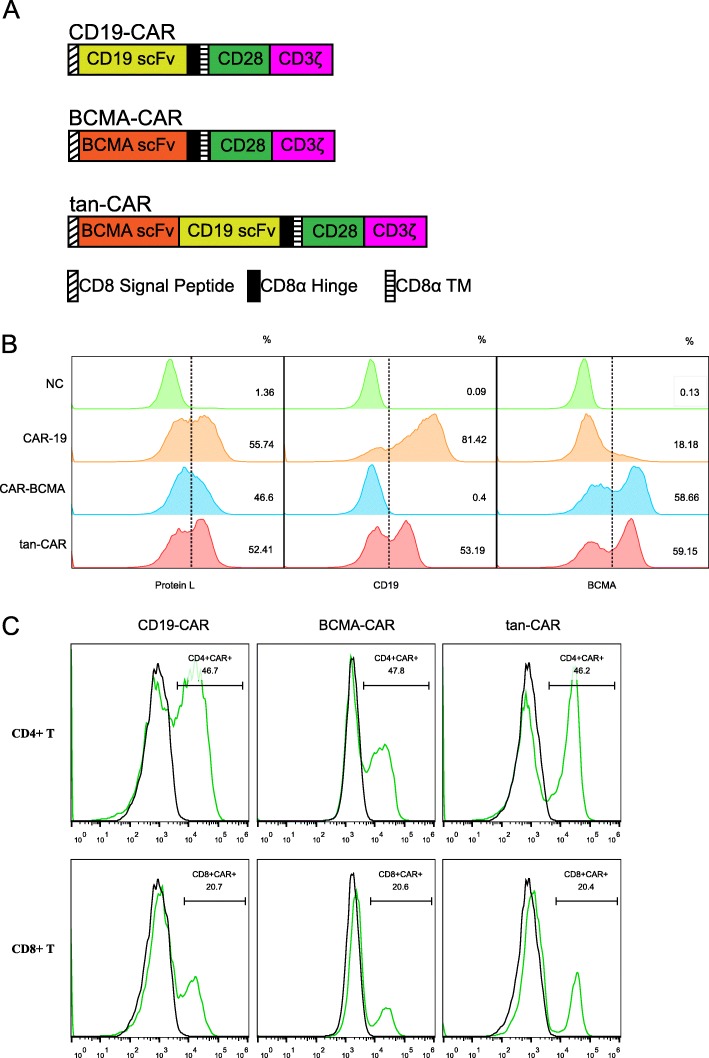
Generation and characterization of tan-CAR T cells. (**a**) Schematic representation of the plasmid design used to construct single and tandem scFv-CAR-modified T cells. The tan-CAR construct encodes a tandem fusion of the BCMA-scFv and CD19-scFv domains, a CD28 costimulatory domain, and a CD3 zeta-mediated activation signaling domain. (**b**) Analysis of transduction efficiency. Isolated and activated human T cells were transduced with the lentiviral vectors CD19-CAR, BCMA-CAR, or tan-CAR. Transduction efficiency was evaluated by flow cytometry using fluorescent-tagged protein L to detect variable light chain Ig together with human CD19 and human BCMA protein. (**c**) Quantitative evaluation of CD4^+^/CD8^+^ T-cell ratios. CAR-positive CD4^+^ T cells and CAR-positive CD8^+^ T cells were detected by flow cytometry. Data are representative of three donors

To develop and to evaluate a CAR T-cell strategy that facilitated simultaneous targeting of both CD19 and BCMA, we designed and generated a novel CAR construct with tandem orientation of both CD19-scFv and BCMA-scFv domains. This tandem-CAR T also included the CD28 costimulatory domain and a CD3ζ-mediated activation signaling component. In parallel, we constructed two control CAR Ts with single scFv domains from anti-CD19 (CD19-CAR) and anti-BCMA (BCMA-CAR) with otherwise identical components (Fig. 1a). Transduction efficiency of the lentiviral vectors that contain the tan-CAR, the CD19-CAR, and the BCMA-CAR targeting primary T cells was evaluated. The surface expression of each of these antigens upon T-cell transduction typically yielded 46 to 55% positive cells; this was verified by flow cytometry using L-protein-FITC to detect cell surface expression of variable light chains (Fig. 1b). Similarly, to validate co-expression of both CD19 and BCMA on tan-CAR-transduced T cells, we introduced tan-CAR effector cells to human CD19 protein or human BCMA protein to define specific CAR T cell detection (for details, see Methods). We found that tan-CAR-transduced T cells expressed both scFvs; 59% were BCMA-scFv-positive and 53% were CD19-scFv-positive; these levels are comparable to those of the single scFv CAR T transductants. Similar results were obtained by flow cytometric detection with FITC-conjugated L protein (Fig. 1b). These results suggested that the tandem fusion of two scFv domains could successfully generate CAR T cells with equivalent expression of both receptor antigens. We also examined the impact of the tan-CAR construct on the CD4/CD8 T-cell ratio (Fig. 1c). As shown, tan-CAR T cells generated a comparable CD4 to CD8 transduced T-cell ratio to those that resulted after T-cell transduction with either CD19-CAR or BCMA-CAR constructs. These results suggest that we might anticipate similar anti-tumor efficacy from all three types of CAR T cells.

### Tan-CAR-transduced T cells are activated by CD19 and BCMA antigens

**Fig. 2:**
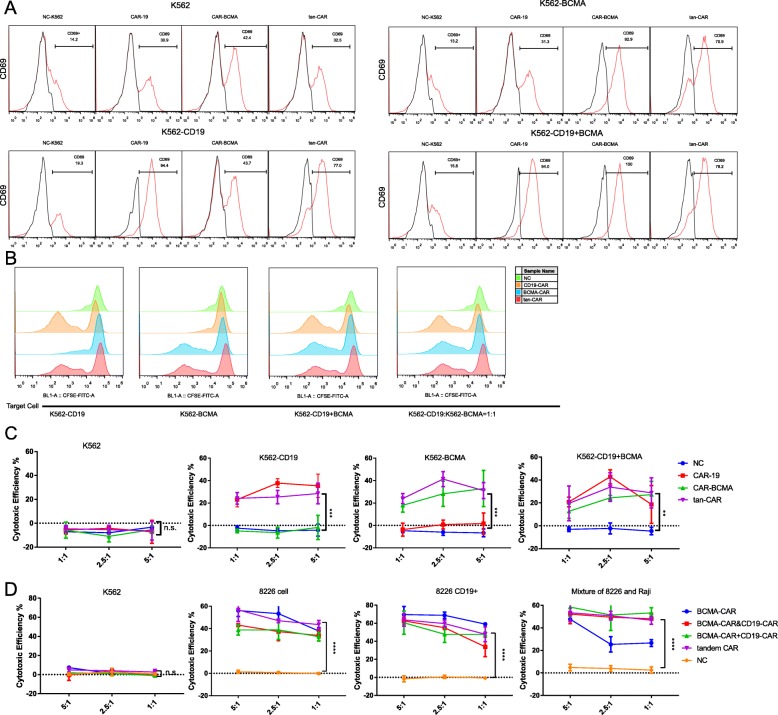
Activation, proliferation and cytotoxicity of Tan-CAR T cells. CAR T-cell activation was evaluated by flow cytometric identification of cell surface expression of CD69. CD69 detected on tan-CAR T cells after co-culture with K562 cells overexpressing CD19, BCMA, or both at levels that were comparable to those of the single scFv-CAR T cells co-cultured with target cells expressing their cognate antigens (*n* = 3). B) Proliferation in response to mitomycin treated K562-transfectant target tumor cells. CAR T cells were labeled with CFSE prior to 5-day co-culture with replication-incompetent target cells. T-cell proliferation was evaluated by flow cytometry. (C)&(D) CAR T-cell-mediated cytotoxicity was evaluated by quantitative assessment of LDH in the supernatants of co-cultured cells as indicated; data are presented as the mean ± SD, *p < 0.05; **p < 0.01; ***p < 0.001 vs. un-transduced T cells (NC) from the same donor

CD69 is a standard marker for T-cell activation [20, 21]. To demonstrate antigen-specific activation of tan-CAR-transduced T cells, cells from the K562 human leukemia line were transfected with lentiviruses encoding CD19 and/or BCMA in order to generate K562-CD19, K562-BCMA, or K562-CD19 + BCMA cells. T-cell activation in response to co-culture with target cells expressed different antigens was analyzed by expression of CD69 on the T-cell surface (Fig. 2a). The tan-CAR T cells responded to both K562-CD19, K562-BCMA, and K562-CD19 + BCMA cells; CD69 expression was detected at levels that were comparable to those observed on activated CD19-CAR and BCMA-CAR T cells. These data indicated that tan-CAR T cells not only express appropriate cell surface antigens but can also be activated by CD19 and BCMA individually or when both are combined. The extent of activation was comparable to that detected among the more conventional single scFv-CAR T cells.

### Tumor cell-induce T-cell proliferation in response to CD19 or BCMA

CAR T-cell proliferation upon recognition of the tumor cell antigen is a fundamental principle and crucial factor underlying the augmented anti-tumor efficacy of CAR T cells [22]. Our next step was to determine whether the proliferation of the tan-CAR T cells was dependent on tumor cell-specific expression of CD19 and/or BCMA.

The three different types of CAR T cells were labeled with CFSE and then placed into co-culture with their corresponding target cells; T-cell proliferation was evaluated via detection of decreasing concentrations of the fluorescent dye by flow cytometry. We found that the tan-CAR T cells underwent extensive proliferation in response to activation by target K562 cells overexpressing CD19 and/or BCMA at an effector: target ratio of 1:1. As anticipated, tan-CAR T cells proliferated to an extent that was similar to those of their corresponding single scFv CAR T positive counterparts. In contrast, the NC (control) T cells underwent only limited proliferation after incubation with the target K562 transfected cells (Fig. 2b). Taken together, these results indicated that both single scFv- and tan-CAR T cells exert significant proliferative activities in response to specific antigen stimulation.

### Determination of the cytotoxic efficacy and specificity of tan-CAR T cells

To evaluate cytolytic specificity of tan-CAR T cells, we first determined the baseline levels of LDH released from target cells over the time course of the experiment. Effector cells including CD19-CAR T, BCMA-CAR T, tan-CAR T, and un-transfected NC T cells were co-cultured with target cells, including K562-CD19, K562-BCMA, K562-CD19 + BCMA and wild-type (un-tranfected) K562 cells as the negative control. After 6 h, significant cytotoxicity was observed in co-cultures of tan-CAR T cells with each of the three transfected K562 target cell groups. As anticipated, CD19-CAR and BCMA-CAR T cells were cytotoxic for their corresponding target cells only; the negative control effector T cells (NC-T) had no cytotoxicity for any of the K562 target cells (Fig. 2c).

We also carried out another experiment to compared the cytolytic efficiency of different effector T cells including: a mixture of CD19-CAR T cells and BCMA-CAR T cells with the ratio of 1:1, the T cells transduced with BCMA-CAR followed by CD19-CAR lentivirus, single scFv-CAR T cells and NC T cells. As for target cells, we use the myeloma cell line of 8226, 8226 cells expressing CD19 antigen and a mixture of 8226 and Raji cells at a ratio of 1:1. After 24 h co-culture, as we expected, the tan-CAR showed great cytolytic efficiency towards all kinds of target cells compared with NC T cells (Fig.2d). No significant differences observed among tan CAR T cells, a mixture of two single scFv T cells and T cells transduced with two lentiviruses. Importantly, BCMA-CAR-T showed slightly less cytolytic ability towards the mixture of 8226 cells and Raji cells compared with tan CAR T cells group, indicating BCMA-CAR T cells were cytotoxic for their corresponding target cells only, but not CD19 antigen positive target cells.

### Determination of CD107a and cytokines release of Tan-CAR T cells

**Fig. 3:**
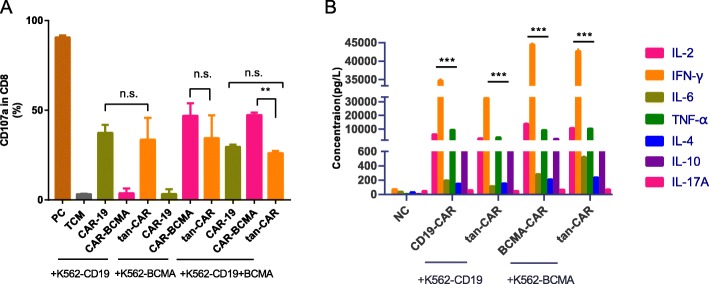
CD107a and cytokines release of Tan-CAR T cells responded to antigen-expressing tumor cells. (**a**) Degranulation of modified T cells was assessed by the appearance of lysosomal protein CD107a on the surface of the CAR T cells in response to co-culture as described. (**b**) Cytokine release in response to co-culture of CAR T cells and target K562 cells as indicated; cytokines evaluated include IL-2, IL-4, IL-6, IL-10, TNF-α, IFN-γ, and IL-17A. Bars represent the mean ± SD of replicate samples. Data are representative of three independent experiments, each with CAR T cells generated from peripheral blood T cells from a different donor

Next, we determined whether cytolytic function was associated with a quantitative increase in the cell surface expression of the lysosomal protein CD107a. After 6-h co-culture with target K562 transfectants, elevated levels of CD107 were detected on tan-CAR T cells compared to levels detected on NC T cells. In parallel, a similar degree of CD107 up-regulation was detected in the CD19-CAR and BCMA-CAR T cells, but only in response to K562 cells expressing their corresponding activating antigen (Fig. 3a).

Finally, proinflammatory cytokine release from CAR T cells was evaluated. We measured cytokine levels in co-culture supernatants using the Th1/Th2 CBA Kit II. As anticipated, the tan-CAR T cells released cytokines to an extent that was comparable to that generated by CD19-CAR and BCMA-CAR T cells in response to their respective cognate antigens. Interestingly, NC-T cells also released proinflammatory cytokines, albeit to a more limited extent, when encountering the target K562 cells (Fig.3b). Collectively, these experiments demonstrated that tan-CAR T-cell-mediated cytotoxicity is equivalent to that of conventional CAR T cells.

### Tan-CAR-transduced T cells are effective at promoting clearance of tumor cells in vivo

**Fig. 4:**
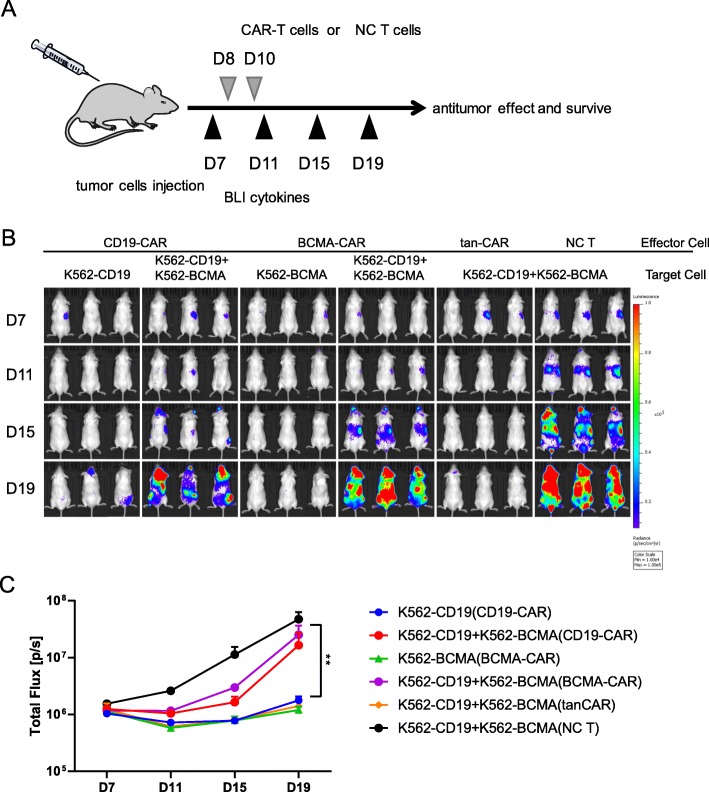
Tan-CAR-transduced T cells effectively clear tumors in vivo. (**a**) Schematic of the xenograft model used to investigate the activity of tan-CAR T cells in vivo. NSG mice were injected with 7 × 10^6^ K562-transduced luciferase-positive target cells via the tail vein; bioluminescent imaging was performed on day 7 and every 4 days thereafter. CAR T cells (10^7^) or NC T cells (10^7^) were provided by intravenous injection on days 8 and 10. (**b**) Bioluminescence radiance was used as a surrogate marker for tumor burden. (**c**) Time course of tumor growth based on mouse whole-body bioluminescence. The mean signal per mouse ± SD is as shown. Statistical analysis was performed using day 19 data (the last time point at which a sufficient number of mice that did not receive treatments remained viable) using one-way ANOVA followed by Tukey’s multiple comparisons test. The data are presented as the mean ± SD (n = 3); ***p < 0.001

We then performed an in vivo study that was designed to evaluate tumor regression in a mouse xenograft model. In this model, NSG mice were injected with target cells including K562-CD19-luc, K562-BCMA-luc, or the mixture of the two target cells at a 1:1 ratio on day 0. These mice were then treated with tan-CAR T cells, CD19-CAR T cells, BCMA-CAR T cells, or NCs on day 8 and day 10 (Fig. 4a) and tumor growth was monitored by IVIS imaging; representative images depicting tumor progression or elimination are shown in Fig. 4b. Among the mice that received either K562-CD19-luc or K562-BCMA-luc target cells, those treated with their respective single scFv-CAR T cells experienced a significant decrease in overall tumor burden based on bioluminescence imaging analysis. However, neither of the single scFv-CAR T cells were capable of inhibiting overall tumor growth in mice with the mixed antigen tumor xenograft. By contrast, tan-CAR T cells showed an efficient anti-tumor inhibitory response and were able to promote tumor regression in all three xenograft models. The signal intensity from IVIS imaging as a function of time is shown in Fig. 4c. These results demonstrated that the conventional single scFV-CAR T cells directed against either CD19 or BCMA were capable of targeting tumor cells that express the corresponding antigens but had no impact on other tumor cells. Most notably, the tan-CAR T cells had a remarkable and specific anti-tumor effect in vivo toward tumor cells that were CD19-, BCMA-positive, or both (*p* < 0.05). Taken together with the results of our in vitro experiments, tan-CAR T cells promote the successful elimination of tumor cells that express either CD19, BCMA, or both; our tan-CAR T cells demonstrate high antigen specificity and in vivo efficacy that is comparable to conventional those of single scFv-CAR T cells.

## Discussion

Adoptive transfer of engineered T cells is a promising approach for the treatment of cancer; unfortunately, post-treatment relapse remains a significant challenge, results suggesting that these therapeutic agents might require further optimization [12]. For example, adoptive transfer of anti-BCMA-CAR T cells to patients with refractory or relapsed MM was initially met with a positive therapeutic response; however, relapse can occur very quickly after the completion of CAR T-cell therapy [12, 23]. This may be related to the absence of the target antigen from the tumor cell surface that has been observed in response to single scFV-CAR T-cell therapy [12]; Previous data showed CD19 expression on plasma cells with characterized cancer stem cell- like properties is a poor prognostic indicator [24]. Clinical data [15] showed BCMA-CAR combined CD19-CAR T cells resulted in MRD negative completely remission leading us to develop tandem CAR-T targeting both CD19 and BCMA, reducing the cost of manufacturing two kind of CAR-T products, while achieving maintaining the long-term remission of patients.

The linkage of two scFvs into a single tandem-CAR in order to generate a bi-specific chimeric receptor has already been proposed in theory [25], although, to the best of our knowledge, this is the first evidence for its successful execution. The tan-CAR enables T cells to recognize two distinct target antigens and to initiate specific killing of the tumor cells that express one or both of the cognate antigens. Perhaps the most critical component of the tan-CAR design is the need to ascertain cytotoxic efficacy that is at least comparable to that of the conventional single scFv-CAR.

To limit the potential for relapse after completion of therapy with BCMA-CAR T cells, we aligned the scFv of anti-CD19 with that of anti-BCMA in a single targeting domain to generate tandem-CAR (tan-CAR). This tan-CAR construct was used successfully to transduce primary human T cells (tan-CAR T cells). In this study, we carefully evaluated the specificity and efficacy of the tan-CAR T in vitro and with respect to a xenograft disease model. Accordingly, this dual scFv CAR construct was activated by either BCMA or CD19 antigens. Moreover, tan-CAR T cells were highly efficacious against antigen-specific tumor cells in both in vitro and in vivo experiments with responses that were comparable to the single scFv-CAR T cells directed against BCMA or CD19. The intrinsic utility of tan-CAR T-cell therapy was also investigated using an immunodeficient mouse model bearing K562-CD19-luc and K562-BCMA-luc target tumor cells. We determined that tan-CAR T-cell specificity and efficacy was fully comparable to those of its single scFv-CAR T counterparts. Notably, tan-CAR enabled T cells to recognize one or both relevant antigens and significantly limited tumor progression in combined target tumor model in mice; by contrast, the tumor burden in the mixed target model increased rapidly in single scFv CAR T-treated mice. Therefore, the bivalent nature of tan-CAR can promote specific T-cell-mediated inhibition of tumor cells that express CD19 and/or BCMA antigen, while the impact of single scFv-CAR T cells was limited to their corresponding single antigen-positive tumor cells.

In summary, we present here the first report of an extensive pre-clinical characterization of novel dual tandem CD19/BCMA-scFv-CAR T cells which were developed for the treatment of refractory or relapsing MM. Our study demonstrated that the tan-CAR which included two specific scFvs from anti-CD19 and anti-BCMA promoted cytolysis of target tumor cells via activation via both CD19 and BCMA. Given the known obstacles to effective CAR T-cell therapy, including absence or loss of BCMA expression, the capacity to target two critical antigens with a single cell infusion could be a promising approach toward addressing problems of refractory disease and disease recurrence. This novel approach might also serve to reduce production costs that would be encountered in providing sequential CAR T-cell treatment for myeloma.

## Conclusion

We report here the design, generation, and evaluation of tan-CAR T cells that can recognize both CD19 and BCMA B-cell antigens, and that can exert significant in vitro and in vivo cytotoxic effects against tumor cells that express one or both of these antigens. This novel and highly effective tan-CAR construct may ultimately be the basis for a novel and effective option for treatment of refractory and recurrent MM, notably among those who have relapsed after effective BCMA-CAR T-cell treatment.

## Data Availability

The data-sets used and/or analyzed during the current study are available from the corresponding author on reasonable request.

## Abbreviations

NC: Untransduced T cell
scFv: Single-chain variable fragment
tan-CAR: Tandem-chimeric antigen receptor
BCMA: B cell maturation antigen
PC: Positive control
TCM: T cell medium
CAR: Chimeric antigen receptor
BCMA: B cell maturation antigen
IL-2: Interleukin 2
IL-4: Interleukin 4
IL-6: Interleukin 6
IL-10: Interleukin 10
TNF-α: Tumor necrosis factor-alpha
IFN-γ: Interferon gamma
IL-17A: Interleukin 17A
NC T: Negative control T cells
